# A Case of Ununited Fracture of the Tibia, of Four Years Standing Successfully Treated

**Published:** 1852-01

**Authors:** W. G. Williams

**Affiliations:** Chillicothe, Ohio


Art. IV.—*A Case of Ununited Fracture of the Tibia, of Four Years Standing successfully treated.* By W. G. Williams, M. D., of Chillicothe, Ohio.

Sometime during last winter, Mr. James Stockton brought to Chillicothe his grand-son, aged four years, to consult me in reference to a deformity of his leg.

The deformity was so great as to render the leg almost useless, and as he informed me, was becoming worse every day.

The previous history of the case as stated to me by Mr. Stockton was, that soon after birth the child appeared uneasy and fretful, crying out on the slightest motion.—On the second day a small lump, which appeared very sore, was discovered on the shin. A physician being consulted, directed them to "let it alone," telling them that it was nothing that would hurt the child. His directions were followed accordingly, nothing being done for the leg, which with very careful nursing, as he said, "got along."

When, at three years of age, the child began to stand on the leg and walk, the limb commenced growing crooked and continued to become worse until the time he consulted with me in reference to it.

On examination I found the tibia so crooked as to form very nearly a right angle, projecting outward and a little forward before the fibula, which was very much curved. So great was the projection, that although three inches above the malleolus, at each step it came within an inch of the floor, the foot resting on its outer edge.

I at once perceived that there was no alternative between straightening the limb and cutting it off, as its condition at that time rendered it worse than a wooden leg. I informed him that it might possibly be straightened by an operation, apprising him however, that the operation was both severe and very dangerous; but nevertheless I considered it worth attempting, under the circumstances, as if (as was much to be feared) we failed to effect an union, we could then resort to amputation, which latter alternative would be best under the circumstances, and far preferable to leaving the limb in its present situation. He left me at that time undecided in the matter, but having subsequently consulted with his friends, he returned in about a month and informed me, that they were willing to submit the child to the proposed operation. Accordingly on the 18th of March, I proceeded to operate, being assisted by Dr. A. C. Johnson who resided in the vicinity, and to whose skilful management and close attention after the operation, I am much indebted for the successful result.

Having put him under the influence of chloroform, I made a longitudinal incision over the projecting angle from two and a half to three inches in length. I then carefully denuded so much of the bone as I intended removing, supposing that I could draw back the integuments so far as to enable me to complete the operation without further incision. In this however, I was mistaken, and found it necessary to make another incision on the outside of the leg, running from the centre of the first, and forming with it a T. After making this last incision, I sawed out the angle without much difficulty, taking care to remove only so much as would permit the bone to be straightened without leaving the fragments too far asunder, and being careful also to let the sections by the saw come together near the under side, without passing quite through, and without wounding or destroying the attachment of that part of the periosteum.

After removing the wedge we found that the parts of the bone could be readily put in apposition, and that no impediment existed to straightening the leg with the exception of the curvature of the fibula, which we were satisfied could be overcome by regularly continued pressure.

This having been done without wounding, or finding it necessary to tie any of the arteries, and consequently with very little hemorrhage, we proceeded to dress the limb as follows:

Having first closed the wound by a few stitches of the interrupted suture, we placed a wet compress upon it, bound it up gently, and then placing a long splint on the inside, and one on the outside of the leg, we secured them above and below the wound by a few turns of the roller. We then put the boy to bed, he being quite comfortable, and having suffered very moderately during the operation.

After remaining with him about two hours, and finding no unpleasant symptoms, I left him in the care of Dr. Johnson, with directions to see him daily, and even twice a day for some time. In consequence of the distance from Chillicothe, I was only able to see him occasionally.

Dr. Johnson has kindly furnished me with a full and minute report of the subsequent symptoms and treatment, from which I make the following abstract:

March 19th.—Slight fever and good appetite. Ordered light diet, and a dose of salts to be given at night.

March 20th.—Has been restless through the night.—Pulse 85. Salts have not moved the bowels. Ordered the salts to be repeated, and Dover's powder to be given at night.

March 21st.—Slept but little. Complains of pain in the knee. Wound suppurating. Pulse 90.

March 22d.—Pulse 80. Wound suppurating freely.—Says he wishes more to eat. Removed one of the stitches and allowed him more generous diet.

March 23d.—This day I saw him, in company with Dr. Johnson. We found much of the wound healed; there being only small openings at the upper and lower ends of the longer incision, and at the junction of the T, which were discharging pus of a good character. Appetite good. Free from fever.

From this time there was little or no variation in the treatment. The suppuration decreased. The splints were readjusted from time to time, and the pressure regularly increased until April 6th, when the stitches having been all removed, and the wound healing well, the starched bandage was applied, to the whole leg and foot, leaving only an opening through which to dress the wound. Over this opening was placed the bandage of Scuttelus, in order that the wound could be dressed without disturbing the position of the bones.

May 2d.—Dr. Johnson reports, wound all healed except a small spot about the size of a pea at the upper edge.

May 8th.—Removed a small piece of bone from the upper edge.

May 21st.—Wound now healed. The bones seem to be solid.

May 31st.—Was called to see the boy, and found him laboring under a severe attack of bilious dysentery. Pulse 104. The limb has the appearance of a large abscess forming under the wound. I removed the starch bandage and reapplied the splints.

June 2d.—I met Dr. Johnson to-day by request. We found the wound open and freely discharging unhealthy pus. Dysentery very slightly improved. Fever high.—Having carefully pressed out all the matter, and applied compresses so as to prevent its accumulation as much as possible, I bound it up. Prescribed for the dysentery acetat. plumb. and opium, &c.

June 9th.—Dr. Johnson reports patient relieved from dysentery, and discharge from wound diminishing.

July 12th.—I saw the boy again to-day, and we removed another piece of bone, which judging from its appearance and other circumstances, I believed to be an isolated portion of the callus, all of which, with the exception of this piece had been absorbed during the fever and dysentery. The leg was now quite limber, and I felt greater fear for the result than I had done at any time previously. However the wound healed readily.

July 20th.—I found the wound entirely healed. We again applied the starched bandage, so as to permit the free motion of the ankle and knee joints, and allowed the boy to walk on the leg after the starch became dry.

Aug. 4th.—Dr. Johnson writes me, "The boy walked half a mile to-day, and does not complain." He says it is as good as the other leg, and again on

Aug. 14th.—He says, "I saw the boy on yesterday; he is well."

Sometime in September, I saw the patient again, and examined the limb carefully and closely. I found the leg as straight as its fellow. The union is now perfect, and the boy walks and runs without the assistance of crutch or stick, and says it is quite well.

He still wears, however, a well constructed wooden boot, divided into two parts, and made to fit so accurately, that when applied they entirely enveloped the leg, forming together a perfect case. We think it best to continue the use of this boot for some time longer as a guard against accidents.

As this operation has been very rarely performed upon the tibia, with success, I have thought proper to report the case, as one encouraging item to those who may be placed under the necessity of deciding for or against an operation under similar circumstances. After consulting all the authorities within my reach, I confess that I found little to encourage me in the undertaking; and had I not been well convinced that matters could not be easily made worse, and entertained at the same time a reasonable hope under all the circumstances, and especially in consideration of the boy's youth and beautiful appearance, that the operation might be attended with success, nothing could have induced me to have made the attempt.

In truth, the boy's beauty and sprightly intelligence had some influence, for I felt that something could and must be done to rescue so fine a boy from so tremendous a mutilation as the loss of a leg.

December 22d, 1851.

